# Supplementary material for: Observing the universal screening of a Kondo impurity
Source: Nat Commun. 2023 Nov 9;14:7263. doi: 10.1038/s41467-023-42857-4 (PMC10636148; doi:10.1038/s41467-023-42857-4)
Supplement: Supplementary file 1 — Supplementary Information [file 41467_2023_42857_MOESM1_ESM.pdf]

Supplementary Information: Observing the universal screening of a Kondo impurity

In this supplementary information, we establish the analytical expressions for $\delta N_{\rm isl}$ and the corresponding susceptibility as presented in Eqs. (4,5) of the main manuscript. Additionally, we provide the non-perturbative expression for $\delta N_{\rm isl}$ and the associated susceptibility in terms of $T/E_{\rm C}$, which is used in Fig. 2b and Fig. 3. Finally, we show and compare with asymptotic predictions the measured charge susceptibility versus transmission probability across the QPC, which provides a complementary view point with respect to $\chi(T)$ displayed in Fig. 3.

I. DERIVATION OF EQS. (4,5) IN MAIN MANUSCRIPT

We consider a metallic island whose electrostatic energy can be controlled thanks to a plunger gate nearby as

$$E_{\rm isl} = \frac{(eN_{\rm isl} - eN_{\rm g})^2}{2C_0}. \tag{1}$$

Here, $N_{\rm isl}$ represents the number of electrons inside the island, $N_{\rm g} = V_{\rm pl}/\Delta$ denotes the normalized gate voltage, and $2C_0$ represents the capacitance of the metallic island. This capacitance also determines the charging energy of the island, which is the energy required to add an electron to it. When $N_{\rm g} = n + \frac{1}{2}$, both the states $N_{\rm isl} = n$ and $n+1$ possess the same energy, thus defining the pseudo-spin of the metallic island. Taking this point as a reference for Kondo mapping by setting $\delta V_{\rm pl} = 0$, the relevant parameter becomes $\delta N_{\rm isl} = N_{\rm isl}(\delta V_{\rm pl}) - N_{\rm isl}(0)$.

The Coulomb blockade phenomenon diminishes as the coupling to an electron bath increases. When the tunnel barrier τ approaches 1, quantum charge fluctuations disrupt the charge quantization, leading to the absence of Coulomb blockade. Thus, in this regime of strong coupling with only one electron bath, a theoretical framework is needed to characterize the state of the island.

Following Matveev[1], we can treat the system composed of the metallic island and the connected quantum Hall channel as one-dimensional. Furthermore, the typical energy scale at which Coulomb blockade becomes significant, denoted by $E_{\rm C}(=1/2C_0)$, is much smaller than the Fermi energy, allowing for the utilization of bosonization techniques. At low energies, the electron system can be regarded as an elastic medium, enabling the definition of two chiral boson fields, $\phi_{\rm u}$ and $\phi_{\rm d}$. The Hamiltonian describing the system with Coulomb interaction and back-scattering becomes $H = H_0 + H_{\rm B}$ where

$$\begin{aligned} H_0 &= \int \frac{dx}{4\pi}\left[(\partial_x\phi_{\rm u})^2 + (\partial_{\rm x}\phi_{\rm d})^2\right] + \frac{E_{\rm C}}{4\pi^2}(\phi_{\rm u}(0) - \phi_{\rm d}(0))^2 - 2E_{\rm C}N_{\rm g}\frac{1}{2\pi}(\phi_{\rm u}(0) - \phi_{\rm d}(0)), \\ H_{\rm B} &= -\frac{Dr}{2\pi}(e^{i(\phi_{\rm u}(0)-\phi_{\rm d}(0))} + e^{-i(\phi_{\rm u}(0)-\phi_{\rm d}(0))}). \end{aligned} \tag{2}$$

Here we set $\hbar = e = k_{\rm B} = v_{\rm F} = 1$. D is the bandwidth of the system, and $r = \sqrt{1-\tau}$ is the back-scattering coefficient. The sign of the back-scattering coefficient is negative following the convention of Eq. (18) in Ref. 1. The bosonic fields satisfy the commutation relations $[\phi_{\rm i}(x), \phi_{\rm j}(y)] = i\delta_{\rm ij}\pi{\rm sgn}(x-y)$. The occupation number of the metallic dot at temperature T, measured from the charge degeneracy point, is obtained from the partition function Z of the system,

$$\delta N_{\rm isl} = \frac{T}{2E_{\rm C}}\frac{1}{Z}\frac{\partial Z}{\partial N_{\rm g}} - \frac{1}{2}. \tag{3}$$

To calculate $\delta N_{\rm isl}$ up to the second order of r, we begin by deriving the partition function using the former boson fields. Initially, we transform these fields into the displacement mode of the elastic medium, denoted as $\phi_{\rm C} = \frac{1}{\sqrt{2}}(\phi_{\rm u} - \phi_{\rm d})$, along with its corresponding momentum mode, $\phi_{\rm I} = \frac{1}{\sqrt{2}}(\phi_{\rm u} + \phi_{\rm d})$. The charge of the island is simply given by the displacement at the position of the constriction, $\phi_{\rm C}(0)$. Consequently, $\phi_{\rm I}$ is decoupled from our physical observable of interest, and we solely consider $\phi_{\rm C}$ in our analysis. The Lagrangian for the $\phi_{\rm C}$ mode is given by $\mathcal{L} = \mathcal{L}_0 + \mathcal{L}_B$, where

$$\mathcal{L}_0 = \frac{v_{\rm F}}{4\pi}\int dx\partial_x\phi_{\rm C}(\partial_t - \partial_x)\phi_{\rm C} - \frac{E_{\rm C}}{2\pi^2}(\phi_{\rm C}(0) - \sqrt{2}\pi N_{\rm g})^2, \quad \mathcal{L}_B = \frac{Dr}{2\pi}(e^{i\sqrt{2}\phi_{\rm C}(0)} + e^{-i\sqrt{2}\phi_{\rm C}(0)}). \tag{4}$$

Note that the electrostatic part (the two last terms of $\mathcal{L}_0$) is now quadratic in bosonic variables and can be treated exactly. Moreover the back-scattering part $\mathcal{L}_B$ has the form of a cosine, leading to a periodic dependence with $N_{\rm g}$.

Fourier transforming the boson field as

$$\phi(x,\tau') = \sqrt{\frac{T}{L}}\sum_{\omega_n,q}\phi(q,\omega_n)e^{iqx-i\omega_n\tau'}, \quad \phi(\tau') = \sqrt{T}\sum_{\omega_n}\phi(\omega_n)e^{-i\omega_n\tau'}, \tag{5}$$

with $\omega_n = 2\pi T n$, $\tau' = it$, and system size L, the partition function becomes

$$Z = \int \mathcal{D}\phi_{\mathrm{C}}(x,\tau')\exp[-\mathcal{S}(\phi_{\mathrm{C}})], \tag{6}$$

where

$$\mathcal{S} = \sum_{\omega_n,q}\frac{q(q-i\omega_n)}{4\pi}\phi_{\mathrm{C}}(q,\omega_n)\phi_{\mathrm{C}}(-q,-\omega_n) + \int_0^{1/T} d\tau' \frac{E_{\mathrm{C}}}{2\pi^2}(\phi_{\mathrm{C}}(0,\tau') - \sqrt{2}\pi N_{\mathrm{g}})^2 + \frac{Dr}{2\pi}(e^{i\sqrt{2}\phi_{\mathrm{C}}(0,\tau')} + e^{-i\sqrt{2}\phi_{\mathrm{C}}(0,\tau')}). \tag{7}$$

To compute δN_{isl}, we expand Eq. (6) in powers of r up to r^2 order, as $Z = Z_0(1 + Z_1 + Z_2 + \cdots)$. At zeroth order of r, the contribution of the gate on the island charge is simply linear, as it is computed from $\frac{1}{Z_0}\frac{\partial Z_0}{\partial N_{\mathrm{g}}} = \frac{2E_{\mathrm{C}}}{T}N_{\mathrm{g}}$. Indeed, at $\tau = 1$ the charge fluctuations completely vanishes and so does the Kondo effect. Up to second order, δN_{isl} can be written as

$$\delta N_{\mathrm{isl}} \simeq \frac{T}{2E_{\mathrm{C}}}\frac{1}{Z_0}\frac{\partial Z_0}{\partial N_{\mathrm{g}}} + \frac{T}{2E_{\mathrm{C}}}\frac{1}{1+Z_1+Z_2}\frac{d(1+Z_1+Z_2)}{dN_{\mathrm{g}}} - \frac{1}{2} \simeq N_{\mathrm{g}} - \frac{1}{2} + \frac{T}{2E_{\mathrm{C}}}\frac{dZ_1}{dN_{\mathrm{g}}} - \frac{T}{2E_{\mathrm{C}}}Z_1\frac{dZ_1}{dN_{\mathrm{g}}} + \frac{T}{2E_{\mathrm{C}}}\frac{dZ_2}{dN_{\mathrm{g}}}. \tag{8}$$

Below we compute Z_1 and Z_2 as a function of N_{g}.

The first order correction, Z_1 is

$$Z_1 = \frac{Dr}{2\pi}\frac{1}{Z_0}\sum_{\zeta=\pm1}\int_0^{1/T} d\tau' \int \mathcal{D}\phi_{\mathrm{C}} \exp\left[\sum_{\omega_n} -\frac{|\omega_n| + E_{\mathrm{C}}/\pi}{2\pi}|\phi_{\mathrm{C}}(\omega_n)|^2 + i\sqrt{2T}\zeta e^{i\omega_n\tau'}\phi_{\mathrm{C}}(\omega_n) + \frac{E_{\mathrm{C}}N_{\mathrm{g}}}{\pi\sqrt{T/2}}\phi_{\mathrm{C}}(0)\right]. \tag{9}$$

First we perform the Gaussian integration over ϕ_{C}. After integrating out ϕ_{C} and τ, we obtain

$$Z_1 = \frac{Dr}{2\pi}\frac{1}{T}\sum_{\zeta}\exp\left[-\sum_{\omega_n}\frac{\pi T}{|\omega_n| + E_{\mathrm{C}}/\pi} - \frac{\pi^2}{E_{\mathrm{C}}}T + 2i\pi N_{\mathrm{g}}\zeta\right] \tag{10}$$

It is important to emphasize that this expression is not limited to small values of T/E_{C}. The temperature can be increased as long as the thermal energy scale remains smaller than the fermi energy. For now we will focus on the regime where $T/E_{\mathrm{C}} \ll 1$ to derive analytical results, but in Sec. II we will evaluate the sum exactly. Then we may replace the following sum by

$$\pi T\sum_{\omega_n}\frac{e^{-|\omega_n|/D}}{|\omega_n| + E_{\mathrm{C}}/\pi} = \log\left(\frac{D}{2\gamma\pi T}\right) - \psi\left(1 + \frac{E_{\mathrm{C}}}{2\pi^2 T}\right) \simeq \log\left[\frac{\pi D}{\gamma E_{\mathrm{C}}}\right] - \frac{\pi^2 T}{E_{\mathrm{C}}} + \frac{\pi^4 T^2}{3E_{\mathrm{C}}^2}; \tag{11}$$

see Eq. (A6b) in Ref. 1. Here $\gamma = e^{\mathbf{C}}$, where $\mathbf{C} \simeq 0.5772$ is the Euler's constant. The partition function therefore is

$$Z_1 = 2\gamma r\cos(2\pi N_{\mathrm{g}})\exp\left[\psi\left(1 + \frac{E_{\mathrm{C}}}{2\pi^2 T}\right) - \frac{\pi^2}{E_{\mathrm{C}}}T\right] \simeq \frac{\gamma r E_{\mathrm{C}}}{\pi^2 T}\cos(2\pi N_{\mathrm{g}})\exp\left[-\frac{\pi^4}{3E_{\mathrm{C}}^2}T^2\right]. \tag{12}$$

This first result gives the first order correction to the linear dependence with N_{g}, recovering the periodic behaviour due to the finite charging energy and the other charge states available.

Next we compute the second order correction Z_2,

$$Z_2 = \frac{1}{2}\frac{D^2r^2}{4\pi^2}\frac{1}{Z_0}\sum_{\zeta_{1,2}=\pm1}\int_0^{1/T} d\tau_1 \int_0^{1/T} d\tau_2 \int \mathcal{D}\phi_{\mathrm{C}}$$
$$\exp\left[\sum_{\omega_n} -\frac{|\omega_n| + E_{\mathrm{C}}/\pi}{2\pi}|\phi_{\mathrm{C}}(\omega_n)|^2 + i\sqrt{2T}(\zeta_1 e^{i\omega_n\tau_1} + \zeta_2 e^{i\omega_n\tau_2})\phi_{\mathrm{C}}(\omega_n) + \frac{E_{\mathrm{C}}N_{\mathrm{g}}}{\pi\sqrt{T/2}}\phi_{\mathrm{C}}(0)\right]. \tag{13}$$

The factor of 1/2 in front originates from the expansion of r^2. After integrating out the ϕ_C field, we obtain

$$Z_2 = \frac{D^2 r^2}{8\pi^2} \sum_{\zeta_{1,2}} \int_0^{1/T} d\tau_1 d\tau_2 \exp\left[\sum_{\omega_n} - \frac{2\pi T\big[1 + \zeta_1\zeta_2 \cos(\omega_n(\tau_1 - \tau_2))\big]}{|\omega_n| + E_C/\pi} - \frac{2\pi^2 T}{E_C}(1 + \zeta_1\zeta_2) + 2i\pi N_g(\zeta_1 + \zeta_2) \right]. \tag{14}$$

Using the same approximation than above for the summation which is valid for $T/E_C \ll 1$ and $0 \ll \tau' \ll 1/T$, it results in

$$\sum_{\omega_n} \frac{2\pi T e^{-|\omega_n|/D}}{|\omega_n| + E_C/\pi} \cos(\omega_n \tau') \simeq -\frac{2\pi^2 T}{E_C} + \frac{2\pi^4 T^2}{E_C^2} \frac{1}{\sin^2(\pi T \tau')}. \tag{15}$$

Now we only consider the N_g dependent part and change the variables as $\tau_a = \tau_1 + \tau_2$ and $\tau_b = \tau_1 - \tau_2$. This transformation comes with a 1/4 Jacobian. Thanks to the former approximation, Z_2 becomes

$$Z_2 \simeq \frac{E_C^2 \gamma^2 r^2}{8\pi^4} \exp\Big[-\frac{2\pi^4 T^2}{3E_C^2} \Big] 2\cos(4\pi N_g) \frac{1}{T} \int_0^{1/T} d\tau_b \exp\Big[-\frac{2\pi^4 T^2}{E_C^2} \frac{1}{\sin^2(\pi T \tau_b)} \Big]. \tag{16}$$

For $0 < \tau_b < \pi/E_C$, or $1/T - \pi/E_C < \tau_b < 1/T$, the integrand becomes negligible. Hence we neglect these parts and focus on $\pi/E_C < \tau_b < 1/T - \pi/E_C$, where the partition function can be approximated by

$$Z_2 \simeq \frac{E_C^2 \gamma^2 r^2}{4\pi^4 T} \cos(4\pi N_g) \exp\Big[-\frac{2\pi^4 T^2}{3E_C^2} \Big] \int_{\pi/E_C}^{1/T - \pi/E_C} d\tau_b \Big[1 - \frac{2\pi^4 T^2}{E_C^2} \frac{1}{\sin^2(\pi T \tau_b)} \Big], \tag{17}$$

Noting that

$$\int_{\pi/E_C}^{1/T - \pi/E_C} d\tau_b \frac{1}{\sin^2(\pi T \tau_b)} = \frac{2\cot(\pi^2 T/E_C)}{\pi T} \simeq \frac{2E_C}{\pi^3 T^2} - \frac{2\pi}{3E_C}, \tag{18}$$

we obtain Z_2 as

$$Z_2 \simeq \frac{E_C^2 \gamma^2 r^2}{8\pi^4 T} \cos(4\pi N_g) \exp\Big[-\frac{2\pi^4 T^2}{3E_C^2} \Big] \Big[\frac{2}{T} - \frac{2\pi^4 T^2}{E_C^2} \Big(\frac{4E_C}{\pi^3 T^2} - \frac{4\pi}{3E_C} \Big) \Big]. \tag{19}$$

Finally, from the derivation of Z_0, Z_1 and Z_2, we can compute the total contribution to the number of electron inside the island up to $(T/E_C)^2$ order and r^2 order (with $r = \sqrt{1-\tau}$ and $N_g = V_{pl}/\Delta$),

$$\delta N_{isl} \simeq \frac{\delta V_{pl}}{\Delta} + \frac{\gamma\sqrt{1-\tau}}{\pi} \Big[1 - \frac{1}{3} \frac{\pi^4 T^2}{E_C^2} \Big] \sin \frac{2\pi \delta V_{pl}}{\Delta} + \frac{2\gamma^2 (1-\tau)}{\pi^2} \Big[1 - \frac{\pi^4 T^2}{E_C^2} \Big] \sin \frac{4\pi \delta V_{pl}}{\Delta}. \tag{20}$$

For $T = 0$ and at the 1st order of r, the above equation coincides with Eq. (25) in Ref 1. This study extended the previously mentioned findings to higher orders of reflections and non-zero temperature, enabling a direct comparison with the experimental data. The charge susceptibility χ at $\delta V_{pl} = 0$ is

$$\frac{\chi}{(g\mu_B)^2} = \frac{\Delta}{2E_C} \frac{\partial N_{isl}}{\partial V_{pl}} \simeq \frac{1}{2E_C} \left[1 + 2\gamma\sqrt{1-\tau} + \frac{8\gamma^2(1-\tau)}{\pi} - \frac{(\pi T)^2}{(E_C/\pi)^2} \Big(\frac{2\gamma\sqrt{1-\tau}}{3} + \frac{8\gamma^2(1-\tau)}{\pi} \Big) \right]. \tag{21}$$

In the main text, this quantity is compared to Kondo predictions. While we observe a T^2 dependence as expected for a Kondo system, it cannot be expressed as a universal function of T/T_K beyond the linear term. This is due to the fact that the Kondo temperature T_K can become quite large as τ approaches one, leading to non-universal contributions beyond the lowest-order in temperature.

II. EXPRESSION FOR FIG. 2B

Here we provide the exact expression of δN_{isl} up to r^2 orders, but valid for arbitrary T/E_C. To obtain the exact result, we use the relations

$$\begin{aligned} 2\pi T \sum_{\omega_n} \frac{e^{-|\omega_n|/D}}{|\omega_n| + E_C/\pi} &= -2\log\Big(\frac{2\gamma\pi T}{D}\Big) - 2\psi\Big(1 + \frac{E_C}{2\pi^2 T}\Big), \\ 2\pi T \sum_{\omega_n} \frac{e^{-|\omega_n|/D}}{|\omega_n| + E_C/\pi} \cos(\omega_n \tau') &= \frac{4\pi^2 T}{E_C + 2\pi^2 T} \mathrm{Re}\left[e^{2i\pi T\tau'} {}_2F_1\left(1, \frac{E_C}{2\pi^2 T} + 1; \frac{E_C}{2\pi^2 T} + 2; e^{2i\pi T\tau'}\right) \right]. \end{aligned} \tag{22}$$

Here ${}_2F_1(a,b;c;z)$ is the hypergeometric function. Therefore $\delta N_{\rm isl}$ is

$$\begin{aligned}\delta N_{\rm isl} =& \frac{\delta V_{\rm pl}}{\Delta} + \frac{2\pi T}{E_{\rm C}}\gamma\sqrt{1-\tau}\exp\Big[\psi\Big(1+\frac{E_{\rm C}}{2\pi^2 T}\Big) - \frac{\pi^2 T}{E_{\rm C}}\Big]\sin\frac{2\pi\delta V_{\rm pl}}{\Delta} \\ &+ \frac{2\pi T}{E_{\rm C}}\gamma^2(1-\tau)\exp\Big[2\psi\Big(1+\frac{E_{\rm C}}{2\pi^2 T}\Big) - \frac{2\pi^2}{E_{\rm C}}T\Big]\sin\frac{4\pi\delta V_{\rm pl}}{\Delta} \\ &\times\left(1 - T\exp\Big[-\frac{2\pi^2}{E_{\rm C}}T\Big]\int_0^{1/T} d\tau'\exp\left[-\frac{2}{1+\frac{E_{\rm C}}{2\pi^2 T}}\mathrm{Re}\Big[e^{2i\pi T\tau'}\,{}_2F_1\Big(1,\frac{E_{\rm C}}{2\pi^2 T}+1;\frac{E_{\rm C}}{2\pi^2 T}+2;e^{2i\pi T\tau'}\Big)\Big]\right]\right).\end{aligned} \tag{23}$$

This is this expression that is used in Fig. 2b of the main text and from which we can extract the susceptibility predictions for Fig. 3.

III. CHARGE PSEUDOSPIN SUSCEPTIBILITY VERSUS TRANSMISSION PROBABILITY

In Fig. 3 of the main manuscript, the charge pseudospin susceptibility is displayed versus temperature, with different symbols corresponding to different tunings of the transmission probability τ of the connected QPC. Here, in Supplementary Fig. S1, we display the same charge susceptibility measurements now as a function of τ with different symbols and colors corresponding to different settings of the temperature up to $T \simeq 69\,$mK. As further described below, the dashed and continuous lines correspond to the asymptotic predictions for our system at small and large τ, respectively.

The theoretical prediction in the strong coupling limit $1-\tau \ll 1$ shown as two continuous black lines in Supplementary Fig. S1 are straightforwardly obtained from $\frac{\chi}{(g\mu_{\rm B})^2} = \frac{\Delta}{2E_{\rm C}}\frac{\partial\delta N_{\rm isl}}{\partial V_{\rm pl}}$ with $\delta N_{\rm isl}$ given in Eq. (23). The lower and higher curves correspond to $T = 9.4\,$mK and $69.7\,$mK, respectively. Note that the comparison is without any fit parameters.

The theoretical predictions in the weak/tunnel coupling limit $\tau \ll 1$ are displayed as dashed lines with a color code corresponding to the indicated experimental temperature, without any free parameters. Following Ref. 2, the plotted predictions of Ref. 3 including perturbative Kondo corrections are given by:

$$\frac{\chi}{(g\mu_{\rm B})^2} = \frac{1}{4k_{\rm B}T}\left(1 - 2\frac{\tau}{4\pi^2}(5.154 + \ln\frac{E_{\rm C}}{\pi k_{\rm B}T})\right) + O[\tau^2, \frac{k_{\rm B}T}{E_{\rm C}}] \tag{24}$$

with $(g\mu_{\rm B})^2 = 2E_{\rm C}$.

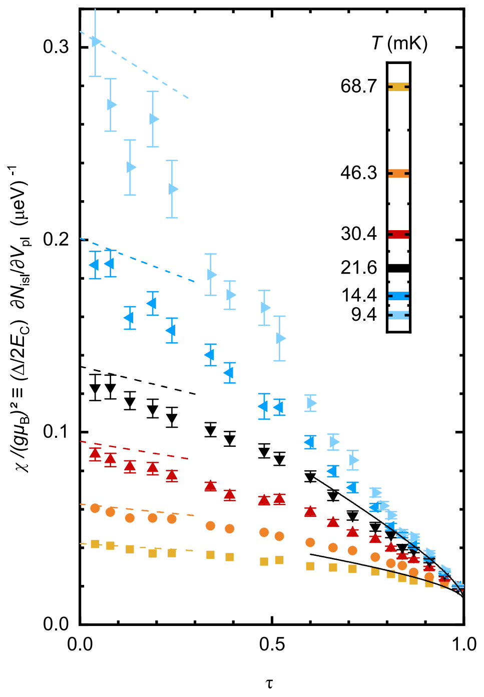

Figure S1. **Charge pseudospin susceptibility versus transmission probability.** The measured charge susceptibility at degeneracy ($\delta V_{\rm pl} \approx 0$) is plotted as a function of the transmission probability τ across the QPC connected to the island. Solid black lines represent the quantitative predictions for the strong-coupling regime ($1 - \tau \ll 1$), computed for both the highest and lowest experimental temperatures (see Supplementary Section III). Dashed colored lines display the quantitative predictions in the weak-coupling/tunnel regime ($\tau \ll 1$) as given by Supplementary Eq. 24 at the experimental temperature indicated by the color code.

[1] K. A. Matveev, Coulomb blockade at almost perfect transmission, Phys. Rev. B **51**, 1743 (1995).

[2] K. W. Lehnert, B. A. Turek, K. Bladh, L. F. Spietz, D. Gunnarsson, P. Delsing, and R. J. Schoelkopf, Quantum charge fluctuations and the polarizability of the single-electron box, Phys. Rev. Lett. **91**, 106801 (2003).

[3] H. Grabert, Charge fluctuations in the single-electron box: Perturbation expansion in the tunneling conductance, Phys. Rev. B **50**, 17364 (1994).
